# A data envelopment analysis of West Virginia school districts

**DOI:** 10.1016/j.heliyon.2019.e01990

**Published:** 2019-07-08

**Authors:** Eduardo Minuci, Amir B. Ferreira Neto, Joshua Hall

**Affiliations:** a John Chambers College of Business and Economics, West Virginia University, PO Box 6025, Morgantown, WV 26506, United States of America; b Lutgert College of Business, Florida Gulf Coast University, 10501 FGCU Blvd. S., Fort Myers, FL 33965, United States of America

**Keywords:** Economics, Data envelopment analysis, Efficiency, Government, Public schools, Education

## Abstract

West Virginia schools are consistently below the national average on the National Assessment of Educational Progress. Using Data Envelopment Analysis, we estimate the technical efficiency of West Virginia school districts. We find less variation in technical efficiency in West Virginia than in similar studies conducted in other states. This appears to be because of state policy imposing homogeneity of input usage. Due to the limited variation in technical efficiency across districts, we cannot analyze how non-school inputs such as socioeconomic factors affect technical efficiency across districts. Summary statistics organized by county economic status, however, suggest that socioeconomic status plays a role. Our results highlight an important limitation of DEA analysis on schools.

## 1 Introduction

Public education focuses on the intellectual and cultural development of human beings. Attending school raises the cognitive skill level of an individual, which positively correlates with economic growth (Hanushek and Woessmann, 2012). Hanushek et al. (2016) estimate that if West Virginia could raise its academic achievement to match the state with the highest education achievement, the state would see over 600% gain in state gross domestic product. Shifting out the education production function is no easy task, especially in a state like West Virginia (WV) that is dealing with persistent budgetary problems due to declining coal severance revenue (Eller, 2017). For any given level of spending, however, ensuring that school districts are operating as close to what is efficient is a way to improve the state's economic situation.

There is a long literature on education production (Hanushek, 1986; Worthington, 2001). In this paper we use data envelopment analysis (DEA) to estimate the technical efficiency of West Virginia School Districts. West Virginia deserves special attention for several reasons. First, the state ranks among the bottom quartile in school rankings (National Education Association, 2017). Second, the state has gained recent national attention with strikes in 2018 and 2019 that spread to other states in the country (Quinn, 2001; Bidgood, 2018; Campbell and West, 2019). Lastly, it is important to understand the academic environment given the state's budgetary problems (Eller, 2017) which should affect school resources and consequently students' performance.

DEA is a mathematical programming approach that identifies the production frontier of a firm (such as a school district) based on existing data and assumptions about the production process (Ruggiero, 2001). Doing so allows us to observe how much inefficiency there currently is in K-12 education in the state. The DEA approach has been used to analyze elementary and secondary schools in many states (Ruggiero and Vitaliano, 1999; Chakraborty et al., 2001; Overton et al., 2016) and countries (Miningou and Vierstraete, 2013; Obadić and Aristovnik, 2011; Aristovnik, 2012; Huguenin, 2015; Munoz and Queupil, 2016; Lauro et al., 2016) as well as institutions of higher education (Calhoun and Hall, 2014; Nazarko and Šaparauskas, 2014).

To preview our results, we find very little variation in technical efficiency across West Virginia school districts. Further investigation highlighted that West Virginia school districts are constrained in terms of input usage by state policy. While this reduces the amount of technical inefficiency, these rules likely constrain districts on the frontier from shifting the education production frontier outward. Due to the limited variation in technical efficiency across districts, we were unable to analyze how non-school input factors affect technical efficiency by district. Summary statistics organized by county economic status, however, suggest that socioeconomic status likely play a role in explaining county-level variation in technical inefficiency.

The remainder of the paper is as follows. Section 2 discusses the DEA approach to measuring technical efficiency and our data on West Virginia county school districts. Section 3 presents our estimates of technical efficiency along with some summary statistics categorized by county economic status. Section 4 concludes with a discussion of our findings and their relevance for West Virginia education policy.

## 2 Methodology

The technical efficiency (TE) numbers drawn from our data envelopment analysis are based on the work done by Bogetoft and Otto (2010).^1^ DEA studies the production process of each county school district every year and determines a measure that represents a 100% efficient system. It then compares the production process of each county school district with the determined standard measure.^2^ This allows the model to calculate a number between zero and one, which qualifies the efficiency of each production process. A school district with a TE equal to one indicates that the county is producing at its maximum level given the choice of inputs it has.

In our research, we consider the Farrel's concept of technical efficiency, in which we assume that a more efficient production process is characterized by producing a certain level of output while utilizing the minimum resources required to do so.^3^ This research's model also assumes the idea of free disposal, determined returns of scale, and convexity of the production possibility frontier. Following Bogetoft and Otto (2010), we can define our model letting $x^{k}$ be the vector of *m* inputs used and $y^{k}$ the *n* outputs produced by firm *k*. The technical efficiency can then be calculated by:

$$TE_{k}=\min_{\begin{array}{c} E,\lambda^{1},...,\lambda^{K} \end{array}}E\;\text{subject to:}\;Ex_{i}^{⁎}\geq \sum_{k=1}^{K}\lambda^{k}x_{i}^{k},\;i=1,...,m\;(I)y^{⁎}\leq \sum_{k=1}^{K}\lambda^{k}y_{j}^{k},\;j=1,...,n\;(II)\lambda \in \Lambda^{K}(\gamma )\;(III)$$

where ⁎ refers to the standard firm, *λ* is the parameter set, and *γ* is an indicator of the return of scale. Bogetoft and Otto (2010) provide further information.

By changing the constraint (III), we can run a test to define whether the production process being analyzed operates at a decreasing or increasing returns to scale. We are able to run this test since the DEA is a non-parametric approach, which does not require us to define a specific production frontier. By solving the system above we are able to calculate a relative measure that represents the geometric distance of each school district's production function from the production possibility frontier (PPF); this generates a measurement bounded between zero and one and represents the technical efficiency of each county of the state. Therefore, we have a relative measure of efficiency.

To calculate the technical efficiency measure, we used the “Benchmarking” package in R described by Bogetoft and Otto (2010). As discussed, we opted to use a input-oriented TE measure, and a variable returns to scale (VRS) set up.^4^ In addition, we estimate the technical efficiency measure for each year individually, which allows for changes in efficiency over time.

The choice of inputs and outputs is very important in the DEA set-up. Hanushek (1986) argues that education is produced with a mixture of school, family, and peer inputs. That is, there are discretionary and non-discretionary variables affecting education. Discretionary variables, are those in direct control by the decision making unit (DMU), in our case the school district. Non-discretionary variables, are those the DMU has no control, and include among others, environmental and socio-economic characteristics, for example. To deal with these different type of variables one can use the so-called multi-stage DEA (Lauro et al., 2016; Simar and Wilson, 2007), or else use these non-discretionary variables as inputs in the TE calculation. In this paper, we opted for the latter approach. We also report TE measures without the non-discretionary variables.

We follow the education production function literature (Miningou and Vierstraete, 2013; Huguenin, 2015; Lauro et al., 2016; Overton et al., 2016) to determine the inputs and outputs for our DEA analysis. In terms of outputs, two measures are usually utilized in the literature: graduation rates and test scores. We use annual state examinations in high school Mathematics and English, along with graduation rates, as our measures of output. While there are many important skills that students learn in school that are not captured on these examinations, the fact that the state examinations and graduation rates are part of the state's school accountability system are a sign that they should be considered primary outputs. During this time frame, West Virginia's state exams (WESTEST 2) ran from grade 3 through grade 11. Given the cumulative nature of education, we use the percentage of 11th graders proficient on WESTEST 2 scores.

In terms of discretionary inputs, the literature focuses on the resources available for the DMU. In this paper, we use expenditures, measured as staff and teachers salary per pupil, and resources available, measured by the number of teacher and staff per pupil. Unfortunately, we do not have data on physical resources such as computers, classrooms, among others. As for non-discretionary inputs, we use the population and real personal income which should account for socioeconomic and cyclical changes. For the non-discretionary variables we use data at the beginning of the school year.

### 2.1 West Virginia school district data

West Virginia has 55 county public school districts. Data on discretionary inputs and output measures for each WV school district for the 2008/2009 to 2014/2015 school years were obtained from the West Virginia Department of Education (WVDOE). Data on the non-discretionary inputs come from the Bureau of Economic Analysis (BEA). Table 1 provides a summary statistics of these variables. Panel A-1 shows the discretionary inputs, Panel A-2 shows the non-discretionary inputs, while Panel B shows the outputs.

**Table 1:** Descriptive statistics for inputs and outputs on DEA.

Statistic	Mean	St. Dev.	Min	Max
**Panel A-1: Discretionary inputs**				
Principal per 100 pupil	0.274	0.068	0.153	0.553
Assistant Principal per 100 pupil	0.133	0.058	0.000	0.333
Teachers per 100 pupil	7.149	0.484	6.038	9.286
Counselor per 100 pupil	0.250	0.052	0.106	0.386
Principal Salary per pupil	24.339	18.022	2.320	73.992
Assistant Principal Salary per pupil	19.583	15.086	0.000	63.142
Teacher Salary per pupil	15.615	11.494	1.514	46.162
Counselor Salary per pupil	16.870	13.109	1.640	58.733

**Panel A-2: Non-discretionary inputs**				
Population (1000s)	33.513	32.952	5.605	193.063
Personal Income (1000s)	28.862	4.614	18.875	41.872

**Panel B: Outputs**				
11th Grade Math Score	0.415	0.145	0.030	1.000
11th Grade English Score	0.432	0.102	0.130	1.000
Graduation Rate	0.820	0.066	0.660	0.970

N=385. Sources: West Virginia Department of Education, Bureau of Economic Analysis.

**Figure 1:**
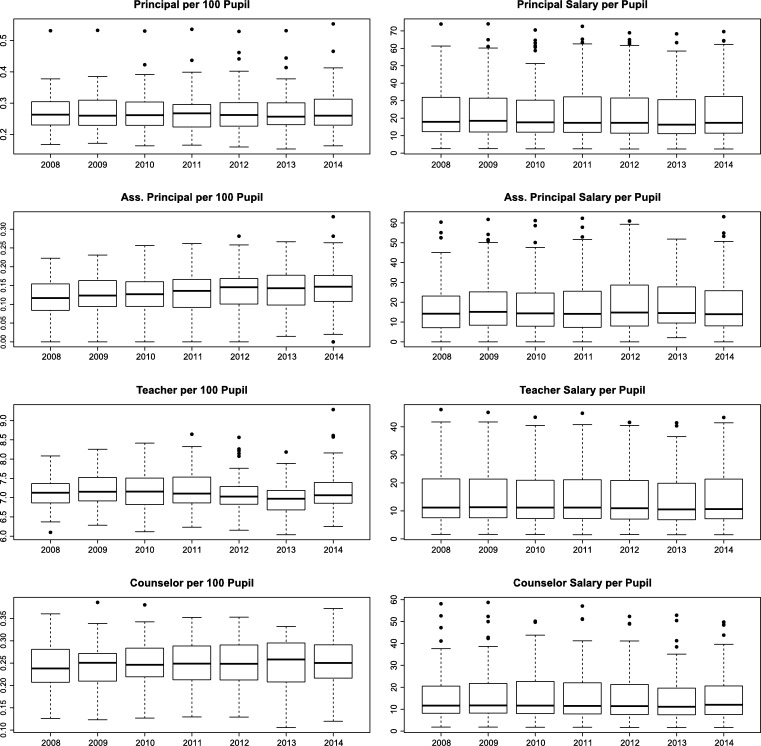
Boxplot of discretionary input variables.

One possible limitation from DEA analysis is the presence of outliers. If this is the case, one possible solution is the use of a super-efficiency analysis (Tørgersen et al., 1996). Fig. 1 illustrates the boxplot for all eight discretionary inputs by year. The boxplots reveal that there are a few outlier observations for some years, hence, indicating that super-efficiency analysis would be preferred. However, when comparing the results from super-efficiency and traditional DEA, the results are identical. Therefore, we proceed our analysis, and report only the traditional DEA results.^5^

## 3 Results

To better understand school district efficiency in West Virginia we create two measures of technical efficiency by combining different inputs and outputs. For both measures of technical efficiency, we use the three listed variable as output, namely, 11th grade test scores in Math and English and graduation rates. For the first technical efficiency measure (TE1) we use only the discretionary variables, i.e., the number of staff members (principals, assistant principals, teachers, and counselor per pupil) and their respective average salaries. As for the technical efficiency measure (TE2), we add the discretionary and non-discretionary variables (income per capita and population). The average technical efficiency results for each West Virginia school district from 2008/2009 to 2014/2015 are presented in Table 2.

**Table 2:** Average technical efficiency by county.

County	AveTE1	AveTE2	County	AveTE1	AveTE2
Barbour	0.97	0.97	Mineral	0.99	1.00
Berkeley	1.00	1.00	Mingo	0.95	0.95
Boone	0.81	0.81	Monongalia	1.00	1.00
Braxton	0.89	0.89	Monroe	0.98	1.00
Brooke	0.99	0.99	Morgan	0.99	0.99
Cabell	1.00	1.00	Nicholas	0.95	0.95
Calhoun	0.90	0.90	Ohio	0.99	1.00
Clay	1.00	1.00	Pendleton	0.96	0.96
Doddridge	0.88	0.88	Pleasants	0.93	0.95
Fayette	0.91	0.91	Pocahontas	0.92	0.92
Gilmer	0.95	0.95	Preston	1.00	1.00
Grant	0.96	0.96	Putnam	1.00	1.00
Greenbrier	0.94	0.94	Raleigh	1.00	1.00
Hampshire	0.99	0.99	Randolph	0.88	0.88
Hancock	0.98	0.98	Ritchie	1.00	1.00
Hardy	1.00	1.00	Roane	0.98	0.98
Harrison	1.00	1.00	Summers	0.93	0.93
Jackson	0.95	0.95	Taylor	1.00	1.00
Jefferson	1.00	1.00	Tucker	0.94	0.94
Kanawha	1.00	1.00	Tyler	0.99	0.99
Lewis	0.97	0.98	Upshur	0.93	0.93
Lincoln	0.89	0.89	Wayne	1.00	1.00
Logan	0.98	0.99	Webster	0.92	0.92
Marion	0.96	0.96	Wetzel	0.95	0.95
Marshall	0.99	0.99	Wirt	0.95	0.95
Mason	0.91	0.91	Wood	1.00	1.00
McDowell	0.95	0.95	Wyoming	0.91	0.91
Mercer	0.97	0.97			

To get a good sense of the overall variation in our data, Table 3 provides summary statistics for our technical efficiency estimates, both under VRS and CRS assumptions. Note that this table included each county school district year measure, not an average like in Table 2. We see very high mean scores across all three output measures, suggesting that on average there is only about 7% technical inefficiency relative to the best performing schools in West Virginia. This suggests that the average West Virginia school district could decrease inputs by 7%, on average, and still keep output (test scores or graduation rates) at the same level.

**Table 3:** Summary statistics for technical efficiency.

Statistic	N	Mean	St. Dev.	Min	Max
TE1-VRS	385	0.960	0.056	0.766	1.000
TE1-CRS	385	0.936	0.074	0.715	1.000

TE2-VRS	385	0.961	0.056	0.766	1.000
TE2-CRS	385	0.938	0.074	0.715	1.000

There are two things to note about the average technical efficiency numbers presented in Table 3. First, there is a consistency across the two efficiency measures. This highlights to us that non-discretionary variables have little influence on the technical efficiency of school districts in West Virginia. Second, there is not a lot of variation in technical efficiency across school districts in the state. The typical mean amount of inefficiency found in these types of studies is in the neighborhood of 20% (Primont and Domazlicky, 2004), with greater variation in technical efficiency across districts.

Salaries are the largest cost of any school district, comprising 80% or more of current expenditures (Myung et al., 2013). That fact, in and of itself, imposes restrictions on input usage by school districts. West Virginia, however, has a state basic salary schedule for teachers. While counties and the state can provide supplements to this base amount for each year in the salary schedule, in practice this has led to much less salary variation across districts than in nearby states. For example, in Ohio the minimum salary for a teacher with no experience and only a BA varies from a minimum of $25,671 in the Southern Local School district to $48,353 in Beachwood City School district (Education Policy Research and Member Advocacy, 2017). In West Virginia, the variation is between $32,675 (several districts) and $36,400 in Monongalia County Schools (West Virginia Department of Education, 2017). This is not surprising given that West Virginia Code states that “the salary potential of school employees employed by the various districts throughout the state does not differ by greater than ten percent between those offering the highest salaries and those offering the lowest salaries.” (WV Code §18A-4-5)

Given that DEA analysis is a relative measure of efficiency, the homogeneity of salaries mandated by West Virginia state law would seem to be leading to the high degree of efficiency in the state. This cost efficiency, however, may come with a downside that cannot be observed in our framework. To the extent that constraints on input usage such as restrictions on compensation, prevent school districts from shifting out the production frontier, West Virginia school districts could be technically efficient but at a lower level of output than could otherwise be achieved. These highlights and important limitation of DEA analyses in education – the legal and institutional environment in which schools operate often determined by state-level policy that affects all observations equally and thus does not directly appear in the analysis.

The concept that imposing budget allocation constraints can limit the ability of school districts to perform well has also been highlighted by Aristovnik (2012) and Overton et al. (2016). Aristovnik (2012) faces similar problems with limitations of input variation; however, the author explores the differences in institutional and legal constraints among countries in the European Union. Overton et al. (2016), on the other hand, focus on the budget constraints imposed by the presence of labor unions, which leads to lower students' performance.

Typically what is done in technical efficiency studies is to regress non-school inputs, such as county demographics, on the measure of technical efficiency. This would be the second stage on a multi-stage DEA analysis. However, in this paper, we opted to use the non-discretionary variables as inputs in the production function, especially in light of the limited degree of demographic variation across school districts.^6^

In Table 4, we provide summary statistics for our technical efficiency measure broken down by Appalachian Regional Commission (ARC) county economic status designation. West Virginia is the only state that lies entirely within the Appalachian region, thus we are able to employ this measure of the persistence of poverty. The ARC uses an index-based classification system to monitor the economic progress of Appalachian counties. The index is based on the comparison of national averages with a three-year average of the unemployment rate, market income per capita, and poverty rate. The ARC then places counties into one of five classifications based on this socioeconomic index: Distressed (bottom 10% ranked counties), At-Risk, Transitional (between 25% and 75% ranked), Competitive, and Attainment (top 10% ranked).

**Table 4:** Technical efficiency by economic status.

Economic Status	N	Mean	St. Dev.	Min	Max
**Panel A: TE1**					
Distressed	71	0.930	0.063	0.793	1.000
At-Risk	114	0.946	0.060	0.766	1.000
Transitional	185	0.976	0.044	0.776	1.000
Competitive	14	1.000	0.000	1.000	1.000
Attainment	1	1.000	NA	1.000	1.000

**Panel B: TE2**					
Distressed	71	0.930	0.063	0.793	1.000
At-Risk	114	0.947	0.060	0.766	1.000
Transitional	185	0.978	0.044	0.776	1.000
Competitive	14	1.000	0.000	1.000	1.000
Attainment	1	1.000	NA	1.000	1.000

Looking at the mean and the min column in Table 4 suggests that counties with higher socioeconomic status seem to be more technically efficient. For example, Competitive and Attainment counties have a mean technical efficiency of 1.00 and a minimum technical efficiency in any one year of 1.00. Contrast that with Distressed counties. While Distressed counties have a mean of 0.93, the minimum technical efficiency is 0.79. In addition to highlighting the importance of socioeconomic status to technical efficiency, these results are also suggestive of the fact that West Virginia school districts in counties that are Competitive or Attainment are constrained at their current level of technical efficiency. Unfortunately, DEA analysis is unable to answer that question.

**Figure 2:**
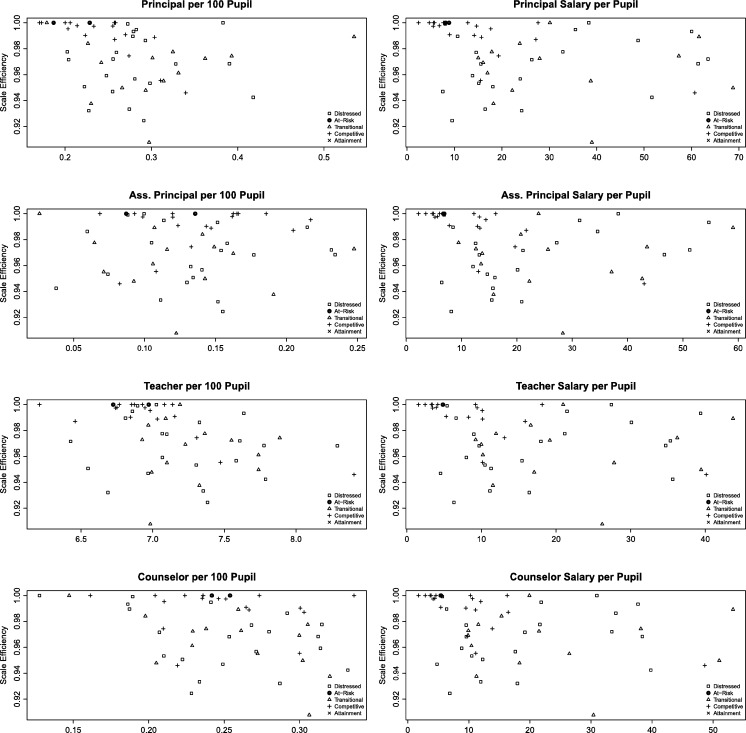
Scale efficiency for TE1.

**Figure 3:**
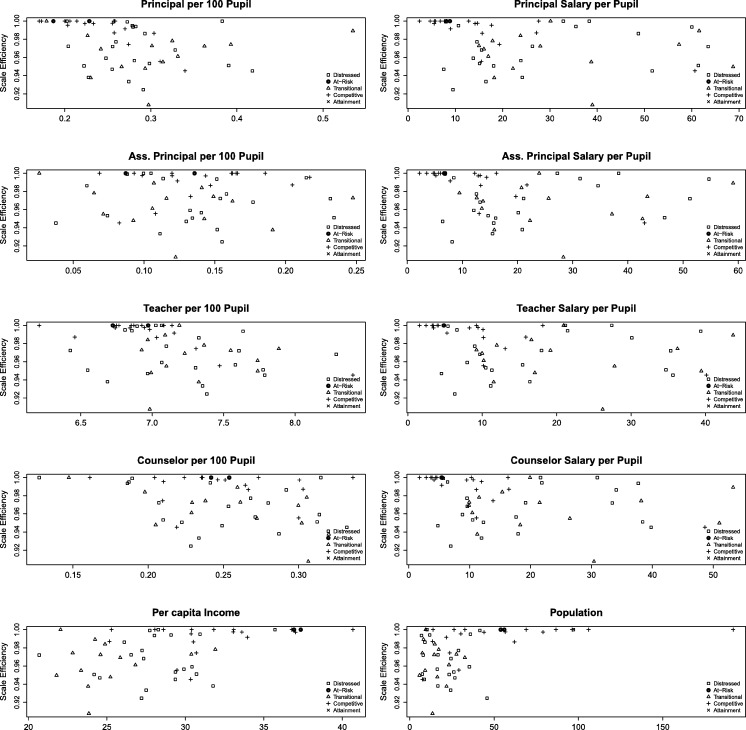
Scale efficiency for TE2.

Lastly, we calculate the scale efficiency (SE) for both technical efficiency measures. Scale Efficiency is defined as the ratio between the technical efficiency under CRS to VRS. According to Bogetoft and Otto (2010) the SE is a measure of closeness to optimal scale size. Fig. 2 reports the average SE for school districts by average economic status for TE1, while Fig. 3 reports it for TE2.

Both Figure 2, Figure 3 corroborate the previous descriptive analysis, such that Competitive counties are closer to their optimal scale. It is interesting to note, that At-Risk counties are also very close to each other, with large SE, which suggest they are operating at their optimal scale. Transitional and Distressed counties however, are very dispersed which is consistent with their struggling economic status. The concept that socioeconomic characteristics can be driving the variation in student performance has also been highlighted by Miningou and Vierstraete (2013) and Huguenin (2015).

## 4 Discussion & conclusions

The primary objective of this paper was to estimate the technical efficiency of West Virginia school districts in order to see if there were cost efficiencies that could be achieved. Our results show that, that the average West Virginia school district is operating at 93% efficiency, well above the average for similar studies. In addition, we see little variation between the level of efficiency among the school districts.

Our findings have two implications for public policy in West Virginia. First, the high level of technical efficiency and the lack of variation reflects homogeneity across school districts. Some of this uniformity is undoubtedly due to the homogeneity in population characteristics across West Virginia counties compared to other settings. On the policy side, however, this uniformity is what appears to be desired policymakers in West Virginia given the requirement that salaries vary no more than 10% across school districts. Our results seem to support that the law is succeeding in leveling the playing field in West Virginia. Second, although the results suggest that education in West Virginia is doing well, this homogeneity might be resulting in a leveling down of education. This would be consistent with cross-state evidence from the National Assessment of Education Progress showing West Virginia schools as consistently being below average.

More generally, our findings highlight an important limitation of DEA analysis. As a relative measure of efficiency, it is only useful to the extent that school districts have the ability to freely use available inputs to shift out the production frontier. However, if school districts or schools are severely constrained, as West Virginia law seems to do by severely restricting teacher salaries, then DEA analysis is of limited use. At a minimum, our results suggest that those utilizing DEA analysis need to carefully consider the legal and institutional context of a locality before interpreting their results.

Given that WV has not shown any signs that this policy which imposes homogeneity in public schools will be modified, social policies are alternatives which could potentially improve students' outcomes as highlighted by Huguenin (2015). For instance, policies which can assist increasing county's entrepreneurship activity, and pre-school, health, housing and unemployment benefits could potentially help counties of lower ARC economic status to economically grow and become more comparable to Competitive and Attainment tagged counties.

Future work could explore the comparison between private and public school, an approach previously explored by Munoz and Queupil (2016), who perform this analysis for the Chilean educational system. Since private schools are not constrained on input allocation as WV public schools are, this extension could potentially shine some light on whether it is the counties' socioeconomic characteristics or the inputs constraint imposed by the state the drivers of school efficiency in WV public school system.

## Declarations

### Author contribution statement

Eduardo Minuci, Amir B. Ferreira Neto, Joshua Hall: Conceived and designed the experiments; Performed the experiments; Analyzed and interpreted the data; Contributed reagents, materials, analysis tools or data; Wrote the paper.

### Funding statement

The authors received no funding from an external source. Within West Virginia University, the authors would like to acknowledge travel funding from the Center for Free Enterprise.

### Competing interest statement

The authors declare the following conflict of interests: Minuci and Neto have no conflicts of interest. Hall has received grants and honoraria totaling more than $10,000 over the past decade from the Charles Koch Foundation, Institute for Humane Studies, Liberty Fund, Texas Tech University's Free Market Institute, Alliance for Market Solutions, and many smaller honoraria for public speaking at universities. A full list is available on his webpage.

### Additional information

Supplementary content related to this article has been published online at https://doi.org/10.1016/j.heliyon.2019.e01990.

No additional information is available for this paper.

## Supplementary material

The following Supplementary material is associated with this article:

DATA combined.xls Raw data used in empirical analysis. DATA combined.xls

## Footnotes

1 Aristovnik (2012) has an excellent discussion of the theoretical underpinnings of this literature. In particular, it is important to note that education production function studies are about relating inputs, such as spending on teachers, to outputs, such as graduation rates. However, the outcome desired out of education might be something entirely different than the measured output, such as future earnings (Hanushek, 1986). Also as noted by Aristovnik (2012), there is a difference between technical and allocative efficiency. Our empirical approach here only focuses on technical efficiency.

2 In West Virginia, school districts match county borders/jurisdiction.

3 There are a number of different methods to approach measuring technical efficiency. A big difference is whether the methods used are parametric or non-parametric (Munoz and Queupil, 2016). The primary difference between the two is that parametric methods have to assume a functional form of the production frontier (Aristovnik, 2012). DEA analyses are typically used in education studies because education often has multiple inputs and outputs (Munoz and Queupil, 2016).

4 We have also calculated the TE measure using a constant return to scale (CRS) assumption and used it to create scale efficiency measures. Nevertheless, the results from VRS and CRS are very similar. The latter is available upon request.

5 The super-efficiency DEA results are available upon request.

6 According to the Census American FactFinder, in 2017, 23% of the WV population is below 20 years-old, there is almost an even split in terms of gender composition and over 93% of the population is white.
